# Negative correlation between IL‐1β, IL‐12 and TNF‐γ, and cortisol levels in patients with panic disorder

**DOI:** 10.1002/brb3.2624

**Published:** 2022-05-19

**Authors:** Ana Belén Fernández‐Serrano, Francisco José Moya‐Faz, Cesar Augusto Giner Alegría, Juan Carlos Fernández Rodríguez

**Affiliations:** ^1^ Health Sciences PhD program Universidad Católica de Murcia UCAM Murcia Spain; ^2^ Chair of Psychogeriatrics Department of Health Sciences Universidad Católica de Murcia UCAM Murcia Spain; ^3^ Department of Legal and Business Sciences Universidad Católica de Murcia UCAM Murcia Spain; ^4^ Director of the Psychology Department Universidad Internacional de la Rioja Logrono Spain

**Keywords:** chronic inflammatory disease, cortisol, depression, gastrointestinal symptoms, intestinal microbiota, proinflammatory cytokines, stress

## Abstract

**Introduction:**

Chronic exposure to stress is a major risk factor in anxiety disorders (ADs) and can be accompanied by an altered microbiome–gut–brain axis and a compromised immune system. In recent years, the study of inflammatory processes in AD has gained special attention. Continued stress causes the reactivity of the hypothalamic–pituitary–adrenal (HPA) axis, the alteration of the intestinal microbiota and the consequent release of pro‐inflammatory cytokines, affecting the sensitivity to stress and the similar behavior of anxiety.

**Method:**

The aim of the present study was to evaluate the interrelationships between measures of proinflammatory cytokines and cortisol in patients with panic disorder (PD).

**Results:**

The main results of the correlation analysis revealed that the levels of pro‐inflammatory cytokines interleukin (IL)‐1β, IL‐12, and tumor necrosis factor gamma were negatively correlated with cortisol scores (area under the curve with respect to the ground).

**Conclusions:**

These results suggest that the inflammatory response is associated with the reactivity of the HPA axis in patients with PD and may influence the maintenance of anxiety behavior.

## Graphical abstract

This study evaluates the interrelationships between pro‐inflammatory cytokine measurements and cortisol in patients with panic disorder.

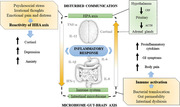

## 1 INTRODUCTION

In recent years, there has been a surprising increase in the research surrounding the role of the microbiome–gut–brain axis (MGBA) in the pathogenesis of anxiety disorders (ADs; Burokas et al., 2015; Dinan & Cryan, 2013; Rea et al., 2019; Tao et al., 2020). The MGBA enables bidirectional signaling between brain and gastrointestinal (GI) function through the central nervous, endocrine, and immune systems, with the intestinal microbiota (IM) being a key influence on a wide range of pathophysiological and psychological processes (Butler et al., 2019).

**FIGURE 1:**
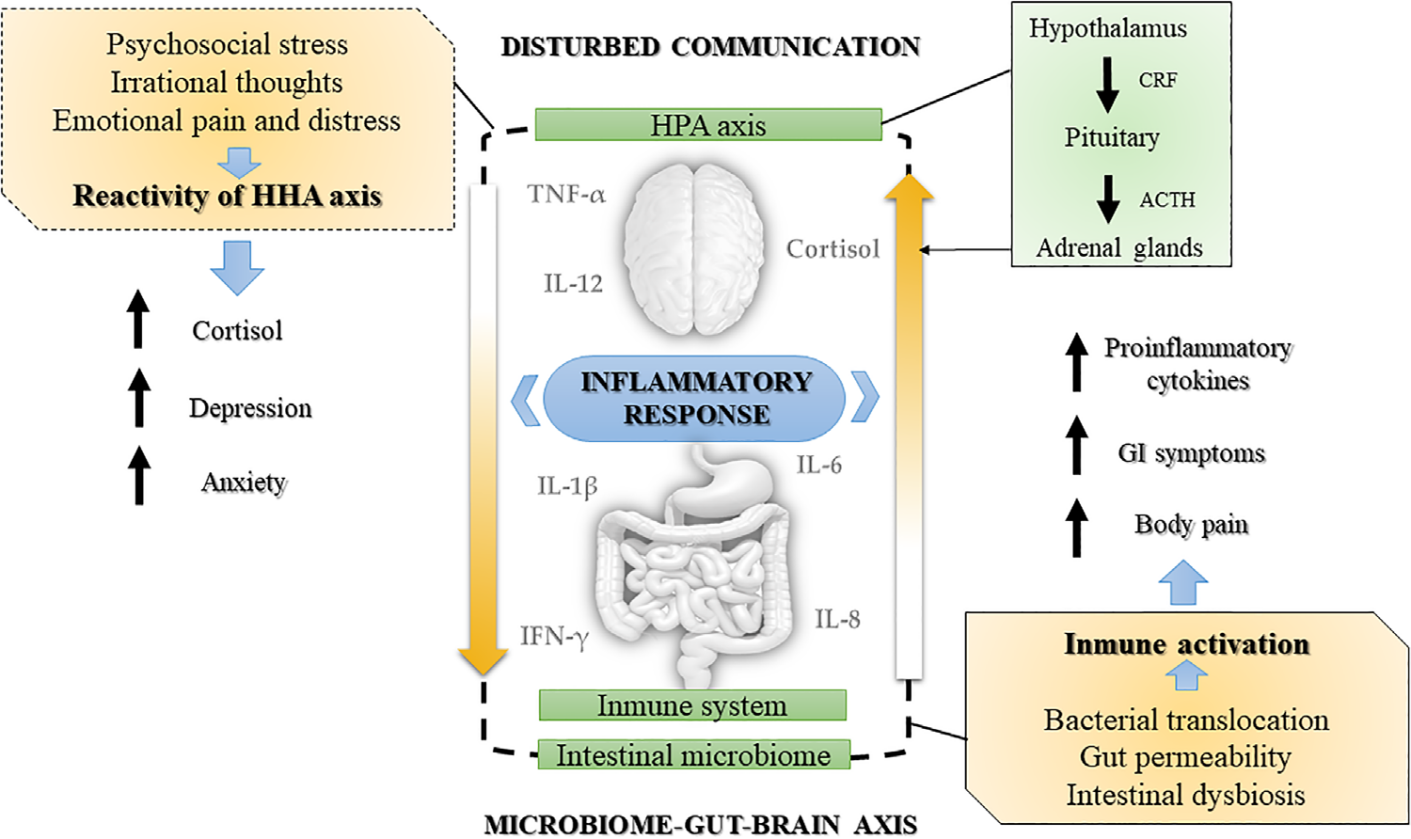
Inflammatory hypothesis of anxiety disorders from the perspective of the microbiome–gut–brain axis (MGBA). *Note*. Chronic stress alters the intestinal microbiota, increasing permeability and the risk of bacterial translocation into the bloodstream. This would trigger an inflammatory response and nonspecific activation of the HPA axis, which would alter the communication of the MGBA. The HPA axis and the immune system are key regulators of this axis. HPA axis, hypothalamic–pituitary–adrenal axis; IL, interleukin; TNF‐α, tumor neurotrophic factor; IFN‐γ, interferon gamma

Current studies have shown that chronic stress can disrupt this extraordinary communication system between the brain and the gut and induce an inflammatory response related to the etiology of anxiety (Peirce & Alviña, 2019). Specifically, the disruption of the physiological pathways of MGBA can contribute to a hyperactivation of the hypothalamic–pituitary–adrenal (HPA) axis with the consequent release of disproportionate levels of cortisol (Forsythe et al., 2010; Foster & McVey Neufeld, 2013). Likewise, the alteration of the MGBA can produce imbalances in the composition of the IM, causing GI alterations (Bonaz et al., 2018) as a result of an intestinal inflammatory response mediated by pro‐inflammatory cytokines. Under normal conditions, IM intervenes in the proper functioning of the HPA axis (Butler et al., 2019), and cortisol influences the maintenance of the immune system, suppressing the expression of pro‐inflammatory cytokines to reduce inflammatory processes (Qing et al., 2020). However, as the systems feed back to each other, maintaining high levels of stress over time can lead to a malfunction of the HPA axis and the release of cortisol. In turn, this may lead to modifications of the composition of IM, which may affect the inflammatory response as well as anxiety and depressive symptoms (Butler et al., 2019; Lange et al., 2020; Manigault et al., 2019; Tao et al., 2020; Wang et al., 2018 ; see Figure 1).

Due mainly to these reasons, the study of inflammation in ADs has gained special interest (Michopoulos et al., 2017; Peirce & Alviña, 2019). Although trials examining links between inflammation and anxiety are less frequent in the literature, clinical research has reported elevated levels of pro‐inflammatory cytokines with increased severity of anxiety symptoms, compared to healthy individuals (Alessi & Bennett, 2020; Niles et al., 2018; Peirce & Alviña, 2019). In particular, patients with panic disorder (PD) present an inflammatory response due to elevated levels of pro‐inflammatory cytokines, such as interferon gamma (IFN‐γ), tumor necrosis factor alpha (TNF‐α), interleukin (IL) 6, and IL‐1β (Alessi & Bennett, 2020; Petrowski et al., 2018; Quagliato & Nardi, 2018). Furthermore, studies have shown that the reduced availability of cortisol under conditions of mental stress in patients with PD could be accompanied by a higher production of IL‐6 and FNF‐α as a latent mechanism of chronic low‐grade inflammation (Petrowski et al., 2018). The severity of panic symptoms seems to have an impact on the inflammatory state, which is seen as a key process that would explain the severity of anxiety symptoms (Schmidt, 2015).

Likewise, AD is also often highly comorbid with other psychiatric disorders, mainly with generalized AD (GAD), PD, agoraphobia, and specific phobias (Michopoulos et al., 2017) as well as with other medical conditions such as chronic inflammatory diseases (CIDs) typical of Westernized countries (Haro et al., 2006; Rook et al., 2014). Among CIDs, epidemiological evidence links ADs to autoimmune disorders (rheumatoid arthritis, systemic lupus erythematosus, inflammatory bowel disease, Hashimoto's thyroiditis, psoriasis) (Alessi & Bennett, 2020; Black et al., 2020; Coit & Sawalha, 2016; Komine, 2020; Ouabbou et al., 2020; Virili et al., 2018), allergic diseases (Yang et al., 2017), and metabolic diseases (hypertension, chronic pain, GI symptoms, dyslipidemia; Belizário et al., 2018; De Gregori et al., 2018; Richards, 2018). These comorbid disorders generate serious consequences on people's health and quality of life (Schnorr & Bachner, 2016; Tao et al., 2020).

Under these circumstances, it is common to relate symptoms of anxiety and depression to higher levels of inflammation as a possible primary mechanism that would explain the prevalence of medical illnesses in people with these psychological symptoms (Niles et al., 2018). Perhaps the breakdown of the correct communication between the intestine and the brain and the associated inflammatory process constitutes a fundamental role between AD, depression, and CID (Amini‐Khoei et al., 2019; Michopoulos et al., 2017; Sarkar et al., 2018). Although the correlations between anxiety and CID are also less studied and more controversial, promoting research on these associations could significantly help establish the clinical importance of EDs in comorbidity with these medical illnesses (Butnoriene et al., 2015; Niles et al., 2018). Some studies show relationships between GAD and the metabolic syndrome mediated mainly by the symptoms of major depression. These facts suggest that both psychiatric symptoms should be taken into consideration when studying metabolic effects (Butnoriene et al., 2015). Another study could not detect an association between anxiety and inflammation but did detect an association with depression and bidirectional results. This means that depressive symptoms induce a worsening of inflammation and that the levels of inflammation increase depressive symptoms (Niles et al., 2018). It is likely that the true etiology of EDs in the presence of depression symptoms comes from MGBA deregulation, which would explain the high levels of comorbidity with these medical illnesses (Ganci et al., 2019).

As presented above, not all studies report results of direct associations between the severity of anxiety symptoms and inflammation, which means that there are multiple confounding variables that make it difficult to obtain consistent results (Jeon et al., 2019). For these reasons, and given that in humans this link is not yet well‐defined (Peirce & Alviña, 2019; Salim et al., 2012), the importance of examining the possible mechanisms involved in AD from a multifactorial approach leads to a unifying conceptualization of health and disease. This would provide a better understanding of these phenomena associated with these highly prevalent disorders: providing new insights into possible causes–effects and for the creation of new study models in the development of innovative and multidisciplinary psychotherapies (Allen et al., 2017; Black et al., 2020; Ganci et al., 2019).

The present study aims to analyze, from an integrative perspective, the interrelationships between the variables associated with the alteration of the MGBA. For this purpose, the following variables will be analyzed: (a) cortisol levels to check the functioning of the HPA axis through the cortisol awakening response (CAR); (b) pro‐inflammatory cytokines and depression levels to study the inflammatory response; (c) GI symptoms as a possible alteration of IM; (d) perceived stress as a psychological response to stressors; (e) and, finally, to check the general physical health status in a clinical sample of patients with PD who attend the public Mental Health services in Spain.

## 2 METHOD

### 2.1 Participants

The sample consisted of 41 patients (*M*: 44.54, standard deviation [*SD*]: 9.42) with an age range between 19 and 64 years. A total of 26.80% were men (*n* = 11), and 73.20% were women (*n* = 30). The patients were under medical care in two centers of the Mental Health network of the Region of Murcia: 58.50% (*n* = 24) came from the Mula Mental Health Center (MHC; Area I Murcia West), and 41.50% (*n *= 17) came from the Caravaca de la Cruz MHC (Area IV Murcia Northwest). They had a diagnosis of PD [F41.0] according to the criteria International Classification of Diseases (ICD) in its tenth version (World Health Organization [WHO], 2000). Nearly all the entire sample presented comorbidity with another AD (80.48%; *n* = 33). A total of 19.51% (*n* = 8) were diagnosed with PD; 21.95% (*n* = 9) PD and mixed anxiety‐depressive disorder (MADD); 9.75% (*n* = 4) PD and GAD; 9.75% (*n* = 4) PD, agoraphobia and MADD; 9.75% (*n* = 4) PD, MADD, and adaptative disorder; 4.87% (*n* = 2) PD and SP; 4.87% (*n *= 2) PD, SP, and MADD; 4.87% (*n* = 2) PD and agoraphobia; 2.43% (*n* = 1) PD and adaptative disorder; 2.43% (*n* = 1) PD, agoraphobia, and social phobia; 2.43% (*n* = 1) PD, GAD, and MADD; 2.43% (*n* = 1) PD, SP, and MADD; 2.43% (*n* = 1) PD, agoraphobia, and adaptative disorder; and 2.43% (*n* = 1) PD, MADD, agoraphobia, and adaptative disorder.

### 2.2 Psychological measures

A semi‐structured interview was developed to study the clinical and sociodemographic characteristics. All participants were evaluated using the following battery of instruments: Perceived Stress Scale (PSS; Remor & Carrobles, 2001) to check the perception of stress; State‐Trait Anxiety Questionnaire (STAI; Buela‐Casal et al., 2015) to measure anxiety intensity and predisposition; Beck Depression Inventory (BDI‐II; Sanz et al., 2005) to classify the severity of depression allowing us to categorize the severity into four groups: “minimal depression” (0 to 13 points), “mild depression” (from 14 to 19 points), “moderate depression” (from 20 to 28 points), and “severe depression” (from 29 to 63 points); Gastrointestinal Symptom Rating Scale (GSRS) (Dimenäs et al., 1995; Kulich et al., 2005) to measure the specific symptoms of GI disorders; and the Short Form 36 Health Survey (SF‐36v2®; Alonso, 1995; Alonso et al., 1998), whose higher scores indicate a better state of health.

### 2.3 Cortisol and pro‐inflammatory cytokine measurements in saliva

Cortisol and pro‐inflammatory cytokine levels were determined by collecting three saliva samples with sterile swabs from the Deltalab brand. To avoid bias in the results due to the impact of covariates, a self‐report sheet with instructions to remove affected data from the analysis was provided. The variables recorded were the following: the time of awakening, the time of saliva collection, the number of hours asleep, the type of awakening (spontaneous or with an alarm clock; Steptoe & Serwinski, 2016), and the exact day of the menstrual cycle in order to avoid the ovulation period, when changes in cortisol levels are produced (Luetters et al., 2007; Stalder et al., 2016).

#### 2.3.1 Cortisol analysis

The reactivity of the HPA axis was determined using the CAR, which expresses the increase in cortisol levels during the first 30–45 min after awakening (Cohen et al., 2019; Powell & Schlotz, 2012; Stalder et al., 2016). Participants were taken a sample upon awakening at 30 and 45 min. They refrained from brushing their teeth, drinking, eating, smoking, and exercising for the duration of the test. After collection, the samples were kept refrigerated until they were taken to the corresponding MHC on that same day. Research personnel collected them and sent them to the laboratory to be frozen at −20°C. On the day of the test, the samples were thawed, vortexed, and centrifuged at 2000–3000 x g for 10 min. Cortisol levels were estimated in microgram per deciliter by the technique enzyme‐linked immunosorbent assay (ELISA) (Thermo Fisher Scientific) with a Meck Millipore reagent kit (Ref. HNCSMAG‐35K) following the manufacturer's instructions. The values of the area under the curve (AUC) were calculated with the three measurements collected as a measure of the CAR (Fekedulegn et al., 2007; Powell & Schlotz, 2012; Pruessner et al., 2003).

#### 2.3.2 Cytokine analysis

IL‐1β, IL‐6, IL‐8, IL‐12, IFN‐γ, and TNF‐α expressed in picogram per milliliter were analyzed. The multiplex immunoassay method was used with the *capture sandwich* technique and the technology Luminex xMAP, with the analyzer Luminex 100/200, the software xPONENT, and the microsphere reagent kit MagPlex 6.5 μm in diameter according to the manufacturer Luminex (Angeloni et al., 2013; Arellano‐Garcia et al., 2008; Bjerre et al., 2009).

### 2.4 Procedure

The clinical sample consisted of individuals with PD who were going to receive group cognitive‐behavioral treatment to control panic and anxiety (Barlow & Craske, 1989). It is a program of 12 weekly sessions of 90‐min duration, based on evidence and empirically validated (Moreno & Martín, 2011). During the first session, the group was presented, evaluated, and consolidated, and the meeting ended with a brief introduction to the cognitive model of panic. The rest of the sessions were dedicated to new strategies for learning and observing the disorder through psychoeducation (Sessions 2 and 3); diaphragmatic breathing and relaxation (Sessions 4–6); cognitive restructuring (Session 7); progressive interoceptive exposure and elimination of escape and avoidance behaviors (Sessions 8–11); and, finally, a review of the knowledge acquired and maintenance for the prevention of relapses was made (Session 12; Barlow & Craske, 1989; Craske & Lewin, 2007; Moreno & Martín, 2011).

Patients belonged to a waiting list derived solely from clinical psychologists and psychiatrists from each respective MHC. During the start of each treatment, the group was offered the possibility of participating in the study. Those who wanted to participate signed up for a list and were summoned to be interviewed and evaluated individually. During the interview, the data were collected, and the scales were administered. If they met the inclusion criteria, the saliva collection kit was delivered, together with the instructions. Saliva samples were collected during the second group session and sent to the laboratory for storage. This protocol was repeated with every group. A total of 47 participants were enrolled, and six were eliminated for not meeting the inclusion criteria. The data were obtained from five groups studied between September 14, 2018, and July 19, 2019. Three groups belonged to the Mula MHC, and two groups belonged to the Caravaca MHC. The inclusion criteria were as follows: (1) meeting the diagnostic criteria for PD [F41.0]; (2) being over 18 years old and under 65 years old; (3) not having other psychiatric disorders (bipolar disorder, hypochondriac disorders, schizophrenia, substance abuse, personality disorder, etc.); (4) does not consume glucocorticoids or other medications that influence the functioning of the immune and endocrine systems; (5) does not have night work shifts that influence the circadian rhythm; (6) does not have or have had serious physical illnesses such as heart disease, cancer, viral infections, or operations of the digestive system; and (7) does not have oral diseases, inflammations, or injuries that can cause bleeding (Stalder et al., 2016).

The study was approved by the ethics committee of the Universidad Católica San Antonio—UCAM, with the code CE041807. Authorization was obtained from the coordinator of Mental Health Areas I Murcia West and IV Northwest and the written informed consent of the participants prior to participation in the study.

### 2.5 Statistical analysis

A descriptive and associative study was carried out to analyze the clinical characteristics and explore relationships between variables. Cronbach's alpha internal consistency coefficient was analyzed for the standardized questionnaires. The chi‐square test was used for the qualitative variables, Student's *t*‐test for age and the clinical variables that fulfilled the assumption of normality, and the Mann–Whitney U‐test was used for the variables that did not show a normal distribution (SF36‐Mental Summation Component (MSC), TNF‐α). A one‐way analysis of variance was performed to check the degree of depression between the clinical and biological variables. Descriptive results and mean differences are expressed as the means (±) and standard deviation (SD). The data were treated with a confidence level of 95% (*p* < .05). The data analysis was developed with JASP statistical software version 0.9.0.1.

## 3 RESULTS

Table 1 shows the clinical characteristics among men and women who attended the MHC with a PD. Neither the quantitative nor the qualitative variables indicated statistically significant differences between men and women. Regarding the reported CID, of the total sample, only 17.10% (*n* = 7) indicated that they did not have a CID. A total of 29.30% (*n* = 12) of the patients reported having allergic diseases, 4.90% (*n* = 2) reported having autoimmune diseases, 9.80% (*n* = 4) reported having metabolic diseases, and 39.10% (*n* = 16) reported concomitance between them (allergic and autoimmune: 19.50%, *n* = 8; allergic and metabolic: 9.80%, *n* = 4; autoimmune and metabolic: 4.90%, *n* = 2; and allergic, autoimmune, and metabolic: 4.90%, *n* = 2).

**TABLE 1:** Clinical characteristics and biological data of the study sample between men and women

	**Men (*n* = 11)**	**Women (*n* = 30)**	***p*‐value** **^a^**
**Sociodemographic characteristics**			
Age, mean (SD)	46.45 (9.17)	43.83 (9.56)	.43^a^
Tobacco use, *n* (%)	4 (36.40)	13 (43.30)	.69^b^
Alcohol consumption, *n* (%)	10 (90.9)	25 (83.30)	.54^b^
Psychotropic drugs, *n* (%)			
Bz	0 (0.00)	6 (20.00)	.10^b^
AD	0 (0.00)	2 (6.70)	
Bz and AD	4 (36.40)	14 (46.70)	
Chronic inflammatory disease, *n* (%)	8 (72.70)	23 (76.70)	.79^b^
**Questionnaire results** (mean ± SD)			
PSS [0–56]	30.55 ± 7.80	34.47 ± 6.88	.12^a^
STAI‐State [0–60]	33.73 ± 12.52	34.43 ± 9.52	.84^a^
STAI‐Trait [0–60]	38.73 ± 11.38	40.53 ± 7.55	.56^a^
BDI‐II [0–63]	20.73 ± 14.41	27.70 ± 10.59	.09^a^
GSRS [7–98]	37.36 ± 17.43	43.50 ± 11.02	.18^a^
SF36‐BP [0–100]	43.38 ± 11.72	37.95 ± 7.83	.09^a^
SF36‐PSC [0–100]	45.90 ± 10.55	46.59 ± 9.16	.84^a^
SF36‐MSC [0–100]	35.99 ± 13.03	30.89 ± 7.43	.22^c^
**Biological variable results** (mean ± SD)			
Cortisol awakening	0.48 ± 0.17	0.49 ± 0.22	.89^a^
Cortisol 30 min	0.47 ± 0.18	0.48 ± 0.21	.94^a^
Cortisol 45 min	0.44 ± 0.18	0.44 ± 0.21	.92^a^
AUCG	21.06 ± 8.03	21.42 ± 9.61	.91^a^
IL‐1β	92.00 ± 21.35	89.47 ± 11.45	.62^a^
IL‐8	77.00 ± 16.60	77.57 ± 13.76	.91^a^
IL‐12	78.18 ± 13.43	75.20 ± 10.22	.45^a^
IL‐6	77.82 ± 11.63	75.83 ± 14.96	.69^a^
FNT‐α	78.64 ± 13.67	75.33 ± 11.17	.36^c^
IFN‐γ	70.00 ± 17.66	66.93 ± 12.61	.54^a^

Abbreviations: AD, antidepressants; AUCG, area under the curve with respect to the ground; BDI‐II, Beck Depression Inventory; BP, body pain; Bz, benzodiazepines; GSRS, Gastrointestinal Symptom Rating Scale; IFN‐γ, interferon gamma; IL, interleukin; MSC, mental summation component; PSC, physical summation component; PSS, Perceived Stress Scale; SF‐36, health questionnaire; SD, standard deviation; STAI, Anxiety Questionnaire (S) State and (T) Trait; TNF‐α, tumor neurotrophic factor.

^a^ Student's *t*‐test for independent samples.

^b^ Chi‐square test.

^c^Mann–Whitney *U*‐test.

Table 2 illustrates the Pearson correlation analysis between all the variables under study. The results show a significant correlation between the psychological and some biological variables. Regarding the psychological variables, the subjects who scored high in stress (PSS) also did so in anxiety (STAI‐S: *r* = .57, *p* < .001; STAI‐T: *r* = .72, *p* < .001), depression (BDI‐II: *r* = .64, *p* < .001), GI symptoms (GSRS: *r* = .35, *p* < .05), and a lower health‐related quality of life (SF36‐BP: *r* = −.40, *p* < 0, 01). The variable IL‐12 correlates negatively with perception of stress (PSS: *r* = −.30, *p* < .05), trait anxiety (STAI‐T: *r* = −.27, *p* < .05), GI symptoms (GSRS: *r *= −.43, *p* < .01), and body pain (SF36‐BP: *r* = −.26, *p* < .05). IFN‐γ negatively correlates with trait anxiety (STAI‐T: *r* = −.35, *p* < .05) and depression (BDI‐II: *r* = −.42, *p* < .01). Finally, IL‐1β also presents an inverse relationship with GI symptoms (GSRS: *r* = −.27, *p* < .05) and body pain (SF36‐BP: *r* = −.31, *p* < .05). In relation to biological variables, cortisol levels (AUC with respect to the ground [AUCG]) show a negative correlation with the pro‐inflammatory cytokines IL‐1β (*r* = −.35, *p* < .05), IL‐12 (*r* = −.30, *p* < .05) and TNF‐γ (*r* = −.30, *p* < .05). IL‐1β has a positive relationship with IL‐8, IL‐12, TNF‐α, and IFN‐γ. IL‐8 positively correlates with IL‐12, IL‐6, TNF‐α, and IFN‐γ.

**TABLE 2:** Correlation matrix between psychological and biological variables

**Variables**	**1**	**2**	**3**	**4**	**5**	**6**	**7**	**8**	**9**	**10**	**11**	**12**	**13**	**14**
1. PSS	–													
2. STAI‐S	**.57** ***	–												
3. STAI‐T	**.72** ***	**.40** **	–											
4. BDI‐II	**.64** ***	**.52** ***	.**56** ***	–										
5. GSRS	**.35** *	**.35** *	**.46** **	**.47** ***	–									
6. SF36‐BP	**−.40** **	**−.29** *	**−.30** *	**−.37** ^**^	**−.67** ***	–								
7. SF36‐PSC	−.21	**−.36** *	−.23	−.19	**−.63** ***	**.78** ***	–							
8. SF36‐MSC	**−.66** ***	**−.47** ***	**−.61** ***	**−.64** ***	**−.38** **	**.36** *	.04	–						
9. AUCG	−.01	−.12	−.06	.17	.22	−.04	.08	−.08	–					
10. IL‐1β	−.17	−.10	−.18	**−.27** *	**−.31** *	.16	.14	.03	**−.35** *	–				
11. IL‐8	−.05	−.00	−.14	−.15	−.27*	.11	.02	.03	−.14	**.77** ***	–			
12. IL‐12	**−.30** *	−.02	**−.27** *	−.24	**−.43** **	**.26** *	.22	.08	**−.30** *	**.57** ***	**.55** ***	–		
13. IL‐6	−.03	.15	.09	−.08	−.25	.11	.09	−.09	−.06	.18	**.39** ^**^	**31** *	–	
14. TNF‐α	.00	.09	−.12	−.08	−.07	−.20	−.15	−.09	−.10	**.41** **	**.41** ^**^	.22	**.35** *	–
15. IFN‐γ	−.20	**−.35** *	−.18	**−.42** **	−.22	.07	.09	.24	**−.30** *	**.60** ***	**.34** *	.25	.07	**.29** *

Abbreviations: AUCG, area under the curve with respect to the ground; BDI‐II, Beck Depression Inventory; BP, body pain; GSRS, Gastrointestinal Symptom Rating Scale; IFN‐γ, interferon gamma; IL, interleukin; MSC, mental summation component; PSC, physical summation component; PSS, Perceived Stress Scale; SF‐36, health questionnaire; SD, standard deviation; STAI, Anxiety Questionnaire (S) State and (T) Trait; TNF‐α, tumor neurotrophic factor.

* *p *< .05;

** *p* < .01;

*** *p *< .00.

Table 3 shows the comparative analysis of the influence of the degree of depression on the psychological and biological variables. The degree of depression has a significant association with the perception of stress, state and trait anxiety symptoms, GI symptoms, and IFN‐γ. Although there are no significant differences in body pain, physical health, AUCG, IL‐1β, and IL‐12, it shows the same pattern conditioned by the degree of depression. Patients with PD who present more severe symptoms of depression are associated with significantly greater anxiety symptoms, GI symptoms, and lower concentrations of IFN‐γ.

**TABLE 3:** Differences in means between the degree of depression and the psychological and biological variables

**Variables**		***n***	***M***	***SD***	***F***	***p***	***η^2^***
PSS	Minimal depression	6	22.67	7.28	**11.07**	**.001**	**0.47**
	Mild depression	9	32.78	4.55			
	Moderate depression	7	32.71	6.62			
	Severe depression	19	37.37	4.81			
STAI‐S	Minimal depression	6	25.50	7.50	**3.23**	**.03**	**0.21**
	Mild depression	9	30.89	8.61			
	Moderate depression	7	35.14	11.45			
	Severe depression	19	38.26	9.62			
STAI‐T	Minimal depression	6	32.00	11.02	**4.22**	**.01**	**0.25**
	Mild depression	9	38.78	8.05			
	Moderate depression	7	37.57	7.39			
	Severe depression	19	44.11	6.51			
GSRS	Minimal depression	6	26.17	8.56	**6.01**	**.002**	**0.33**
	Mild depression	9	38.00	7.51			
	Moderate depression	7	47.43	8.58			
	Severe depression	19	46.58	13.65			
SF36‐BP	Minimal depression	6	48.09	11,93	2.42	.08	0,16
	Mild depression	9	39.32	5,93			
	Moderate depression	7	38.04	9,47			
	Severe depression	19	37.21	8.50			
SF36‐PSC	Minimal depression	6	52.43	5.26	1.07	.37	0.08
	Mild depression	9	46.90	6.75			
	Moderate depression	7	44.92	5.44			
	Severe depression	19	44.81	11.98			
AUCG	Minimal depression	6	15.90	9.88	1.85	.14	0.13
	Mild depression	9	18.77	10.27			
	Moderate depression	7	26.34	3.85			
	Severe depression	19	22.40	9.03			
IL‐1β	Minimal depression	6	93.67	13.52	1.45	.24	0.10
	Mild depression	9	97.33	11.31			
	Moderate depression	7	89.43	10.55			
	Severe depression	19	85.89	16.54			
IL‐12	Minimal depression	6	81.83	9.41	2.10	.11	0.15
	Mild depression	9	81.11	11.50			
	Moderate depression	7	74.43	10.40			
	Severe depression	19	72.32	10.65			
INF‐γ	Minimal depression	6	78.17	17,42	**2.73**	**.05**	**0.18**
	Mild depression	9	70.67	12,88			
	Moderate depression	7	70.71	13,62			
	Severe depression	19	62.00	11.63			

Abbreviations: AUCG, area under the curve with respect to the ground; BDI‐II, Beck Depression Inventory; BP, body pain; GSRS, Gastrointestinal Symptom Rating Scale; IFN‐γ, interferon gamma; IL, interleukin; MSC, mental summation component; PSC, physical summation component; PSS, Perceived Stress Scale; SF‐36, health questionnaire; SD, standard deviation; STAI, Anxiety Questionnaire (S) State and (T) Trait; TNF‐α, tumor neurotrophic factor.

## 4 DISCUSSION

To our knowledge, this is the first study to evaluate, from the integrative perspective of the MGBA, the association between levels of pro‐inflammatory cytokines (IL‐1β, IL‐8, IL‐12, IL‐6, TNF‐α, and IFN‐γ), morning cortisol levels and psychological variables in patients with PD. This is because stressors could lead to MGBA symptoms, creating a depletion of the physiological stress and immune response. Over time, it would trigger an *intestinal vulnerability* with GI and inflammatory consequences and a *psychological vulnerability* with dysfunctional cognitive patterns that would influence the sensitivity of stress and the similar behavior of anxiety and depression, with a feedback character (Giollabhui et al., 2020; Li et al., 2019; Zainal & Newman, 2022).

To begin with, our findings showed that PD patients who interpreted life circumstances with a greater sense of insecurity and uncontrollability, scoring high in perceived stress, presented higher symptoms in both state and trait anxiety, depression, GI symptoms, body pain, and worse physical health. These facts showed a direct association between the variables. It seems that patients with PD show characteristic symptoms that could be related to the alteration of the MGBA. This is in line with the accumulated evidence showing that bidirectional dysregulation between the gut and the brain, responsible for connecting emotional and psychological centers with GI functions (Dinan & Cryan, 2013; Johnson et al., 2020; Sarkar et al., 2018), are associated with higher anxiety and depression scores, physical symptoms such as GI discomfort, and an increased susceptibility to visceral pain (Black et al., 2020; Takada et al., 2016), possibly attributable to stressful events (Takada et al., 2016). Previous studies have shown that it is common in those patients who present body pain and bothersome GI symptoms to be a consequence of a chronic inflammatory state (Belizário et al., 2018). Perhaps for this reason, according to our analysis, we found that, in addition to anxiety and depression, GI symptoms, body pain, and a more deteriorated state of health were related to each other in patients with PD. This new integrating concept of the physiology of the organism guarantees the correct functioning of the intestine through visceral messages, which exert profound effects on the regulation of stress and in the similar behavior of anxiety and depression (Allen et al., 2017; Cryan et al., 2019; Foster et al., 2017; Mayer, 2011). Any threat that alters the MGBA can change the appropriate functioning of both distal organs (Khlevner et al., 2018) and produce an inflammatory response implicated in the pathophysiology of ADs and a wide range of chronic medical conditions (Meuret et al., 2020; Michopoulos et al., 2017).

In order to verify the interactions between the HPA axis and the inflammatory response in patients with PD, we observed during our study that cortisol levels were negatively correlated with the concentrations of pro‐inflammatory cytokines IL‐1β, IL‐12, and IFN‐γ. This means that higher levels of IL‐1β, IL‐12, and IFN‐γ were linked with a less pronounced CAR and vice versa. This was consistent with previous findings (Petrowski et al., 2018), which would suggest that there is a link between the HPA axis and the immune system in PDs. Both the HPA axis and the immune system are part of the MGBA signaling mechanisms, and both systems influence each other and are orchestrated by the IM, being able to alter the inflammatory response and promote various physical and mental disease processes (Manigault et al., 2019; Petrowski et al., 2018). Other studies indicate that gut bacteria are implicated in virtually all noncommunicable diseases as a result of immune dysregulation, altered stress response, and lifestyle factors (Butler et al., 2019). It should be noted that IM communicates with the brain through cytokines released from the intestinal mucosa, which are responsible for modulating the body's inflammatory response. These proteins travel to the brain by crossing the blood–brain barrier or through the metabolism of neurotransmitters, activating the vagus nerve or the HPA axis (Alessi & Bennett, 2020; Butler et al., 2019; Johnson et al., 2020; Raff & Levitzky, 2013).

In this regard, our results could suggest that the reactivity of the HPA axis would lead to an ineffective inflammatory response of the organism with consequences in affective behavior (Lopresti, 2017; Michopoulos et al., 2017; Milrad et al., 2018). In fact, psychological variables were associated with some pro‐inflammatory cytokines. IL‐12 was negatively correlated with perceived stress, trait anxiety, GI symptoms, and body pain. The same happened with IFN‐γ, which was inversely correlated with trait anxiety and depression. IL‐1β was negatively related to GI symptoms and body pain in patients with PD. In this sense, the greater the severity of the symptoms of the psychological variables, the lower the levels of pro‐inflammatory cytokines. This finding is in line with research like that of Tükel et al. (2012), who found a relationship between reduced levels of IL‐12, IFN‐γ, and TP. This indicates that having inadequate levels of IFN‐γ could have a negative impact on psychological stress and be a cause of anxiety since it intervenes in the normal activity of the amygdala and influences the regulation of the HPA axis. Furthermore, it is important to note that fluctuations in the reactivity of the HPA axis and pro‐inflammatory cytokines in patients with PD may be dependent on the severity of depression (Butnoriene et al., 2015; Niles et al., 2018). This was reflected in the analysis of mean differences between the degree of depression and the variables measured. On the one hand, both perceived stress, state and trait anxiety, GI symptoms, and cortisol (although the latter was not significant) showed higher scores depending on the severity of depression. On the contrary, the more severe the depression, the lower the IFN‐γ levels, and similarly, although they were not significant, IL‐1β and IL‐12 presented the same pattern. It seems that depending on the degree of depression suffered by patients with PD, cortisol and cytokine concentrations fluctuate inversely. That is, the greater the depression is, the higher the levels of anxiety and cortisol and the lower the levels of IFN‐γ, IL‐1β, and IL‐12. These results could agree with the research by Butnoriene et al. (2015), who showed an association between GAD and the metabolic syndrome mediated by the symptoms of major depression. Other research found that elevated CAR can predict depression (Fiksdal et al., 2019; Pruessner et al., 2003; Steptoe & Serwinski, 2016) and is related in subjects with anxiety symptoms in comorbidity with depression (Vreeburg et al., 2010). In another study, Petrowski et al. (2018) reported that the reduced availability of cortisol under conditions of psychological stress in patients with PD may be accompanied by an increased production of pro‐inflammatory cytokines as a latent mechanism of chronic low‐grade inflammation. Other authors have observed the same reversed effect between the diurnal pattern of cortisol and pro‐inflammatory cytokines (IL‐1β, IL‐12, TNF‐α, and IFN‐γ; Petrovsky et al., 1998; Yang et al., 2017). Nevertheless, this does not mean that cortisol can inhibit cytokine production but that glucocorticoids could neutralize the transcription factor of some cytokines (IL‐1, IL‐6, IL‐8, IFN‐γ) by negatively regulating them. Cortisol is likely to interfere with the balance between Th1 and Th2 cells by preventing the release, for example, of IFN‐γ produced by Th1 cells (Petrovsky et al., 1998). In any case, and given that the underlying mechanisms are not clear, according to our results, the pattern of variation found between the levels of pro‐inflammatory cytokines and cortisol may be conditioned by the degree of depression that patients with PD have.

Likewise, it is important to note that most of the patients who attended mental health with a PD (83%, *n* = 34) reported having a CID, whether it was allergic, autoimmune, metabolic, and/or combined with each other. This could be because increased inflammation can also have detrimental consequences on physical health (Michopoulos et al., 2017). Our data agree with the idea that increased ADs are generally concomitant with these chronic inflammatory disorders, intrinsically related to lifestyle. For this reason, the alteration of the MGBA and the associated inflammatory process could be the link that links the CID, the AD, and depression (Alessi & Bennett, 2020; Michopoulos et al., 2017; Paiva et al., 2020; Schnorr & Bachner, 2016). In short, if anxiety is the cause of the inflammatory response or if inflammation causes an increased anxiety response and this is dependent on the symptoms of depression, it is a question to be confirmed since there are multiple confounding variables that prevent obtaining robust results (Jeon et al., 2019). However, it must be considered that the dysregulation of the MGBA could sustain the inflammatory response from becoming ineffective and contribute over time to the increase in anxiety levels and, perhaps, interfere with the maintenance and worsening of symptoms (Jeon et al., 2019; Michopoulos et al., 2017). For these reasons, and in line with the biopsychosocial model that understands the disease from a multifactorial point of view, considering the existence of a powerful connection between the brain and the intestine would help explain the variables associated with PDs and provide a new conceptualization for understanding possible causes (Black et al., 2020). Also, it would promote new diagnostic evaluation methods and evaluate the administration of adjuvant treatments to improve the effects of established first‐choice interventions in PDs (Petrowski et al., 2018).

Despite the relationships found, the present study had some limitations. In addition to a small sample size, it did not consider influencing lifestyle variables such as type of diet, pharmacology, exposure to trauma, physical exercise, antibiotics, and so forth. Similarly, the results do not allow conclusions to be drawn about the levels of cytokines and cortisol in patients with PD. In future studies, longitudinal research is needed to analyze the existence of common inflammatory mechanisms between AT and CID that can explain the causes. It would be interesting to analyze the bidirectionality of appearance over time of GI and psychological symptoms (Black et al., 2020) and to study the moderating effects of biopsychosocial variables associated with the inflammatory and endocrine response in patients with PD. Additionally, the standardization of the covariates that influence inflammation was performed to facilitate comparisons between studies (Majd et al., 2020).

In conclusion, our findings provide direct evidence that, in addition to perceived stress, anxiety, and depression, PD patients have more severe GI symptoms, body pain, and more impaired physical health. This relationship is probably mediated by the negative correlation between the reactivity of the HPA axis and pro‐inflammatory cytokines IL‐1β, IL‐12, and IFN‐γ. Furthermore, some pro‐inflammatory cytokines (IL‐12, IFN‐γ, and IL‐1β), but not all, are involved in the regulation of anxiety in patients with PD and are negatively associated with greater severity of depression, GI symptoms, bodily pain, and poorer physical health. It is likely that the fluctuations found between the reactivity of the HPA axis and the cytokines may be dependent on the degree of depression suffered by patients with PD. In this sense, the greater the depression in patients with PD, the higher the state and trait anxiety, the perceived stress, the GI symptoms, the reactivity of the HPA axis and the lower the levels of IFN‐γ, IL‐1β, and IL‐12. This could be consistent with the idea that the alteration of the bidirectional communication between the MGBA and the associated inflammatory response may be mediating both the severity of the psychological symptoms and the physical symptoms involved in PD.

## CONFLICT OF INTEREST

The authors have no competing interests and have published this article at the stage of preparation.

### PEER REVIEW

The peer review history for this article is available at https://publons.com/publon/10.1002/brb3.2624.

## Data Availability

The data that support the findings of this study are available from the corresponding author.

## References

[brb32624-bib-0001] Alessi, M. G. , & Bennett, J. M. (2020). Mental health is the health of the whole body: How psychoneuroimmunology & health psychology can inform & improve treatment. Journal of Evaluation in Clinical Practice, 26(5), 1539–1547. 10.1111/jep.13386 32171052

[brb32624-bib-0002] Allen, A. P. , Dinan, T. G. , Clarke, G. , & Cryan, J. F. (2017). A psychology of the human brain–gut–microbiome axis. Social and Personality Psychology Compass, 11(4), e12309. 10.1111/spc3.12309 28804508 PMC5530613

[brb32624-bib-0003] Alonso, J. (1995). La versión española del SF‐36 Health Survey (Cuestionario de Salud SF‐36): Un instrumento para la medida de los resultados clínicos. Medicina Clínica, 104, 771–776. 7783470

[brb32624-bib-0004] Alonso, J. , Prieto, L. , Ferrer, M. , Vilagut, G. , Broquetas, J. M. , Roca, J. , Batlle, J. S. , & Antó, J. M. (1998). Testing the measurement properties of the Spanish version of the SF‐36 Health Survey among male patients with chronic obstructive pulmonary disease. Journal of clinical epidemiology, 51(11), 1087–1094. 9817126 10.1016/s0895-4356(98)00100-0

[brb32624-bib-0005] Amini‐Khoei, H. , Haghani‐Samani, E. , Beigi, M. , Soltani, A. , Mobini, G. R. , Balali‐Dehkordi, S. , Haj‐Mirzaian, A. , Rafieian‐Kopaei, M. , Alizadeh, A. , Hojjati, M. R. , & Validi, M. (2019). On the role of corticosterone in behavioral disorders, microbiota composition alteration and neuroimmune response in adult male mice subjected to maternal separation stress. International Immunopharmacology, 66, 242–250. 10.1016/j.intimp.2018.11.037 30500621

[brb32624-bib-0006] Angeloni, S. , Cordes, R. , Dunbar, S. , Garcia, C. , Gibson, G. , Martin, C. , & Stone, V. (2013). *xMAP® Cookbook. A collection of methods and protocols for developing multiplex assays with xMAP Technology*. Luminex xMAP Technology. www.luminexcorp.com

[brb32624-bib-0007] Arellano‐Garcia, M. , Hu, S. , Wang, J. , Henson, B. , Zhou, H. , Chia, D. , & Wong, D. (2008). Multiplexed immunobead‐based assay for detection of oral cancer protein biomarkers in saliva. Oral Diseases, 14(8), 705–712. 10.1111/j.1601-0825.2008.01488.x 19193200 PMC2675698

[brb32624-bib-0008] Barlow, D. H. , & Craske, M. G. (1989). Mastery of your anxiety and panic. Graywind Publications.

[brb32624-bib-0009] Belizário, J. E. , Faintuch, J. , & Garay‐Malpartida, M. (2018). Gut microbiome dysbiosis and immunometabolism: New frontiers for treatment of metabolic diseases. Mediators of Inflammation, 2018, 1–12. 10.1155/2018/2037838 PMC6304917 30622429

[brb32624-bib-0010] Bjerre, M. , Hansen, T. K. , Flyvbjerg, A. , & Tønnesen, E. (2009). Simultaneous detection of porcine cytokines by multiplex analysis: Development of magnetic bioplex assay. Veterinary Immunology and Immunopathology, 130(1‐2), 53–58. 10.1016/j.vetimm.2009.01.007 19230983

[brb32624-bib-0011] Black, C. J. , Drossman, D. A. , Talley, N. J. , Ruddy, J. , & Ford, A. C. (2020). Functional gastrointestinal disorders: Advances in understanding and management. The Lancet, 396(10263), 1664–1674. 10.1016/S0140-6736(20)32115-2 33049221

[brb32624-bib-0012] Bonaz, B. , Bazin, T. , & Pellissier, S. (2018). The vagus nerve at the interface of the microbiota‐gut‐brain axis. Frontiers in Neuroscience, 12, 49. 10.3389/fnins.2018.00049 29467611 PMC5808284

[brb32624-bib-0013] Buela‐Casal, G. , Guillen‐Riquelme, A. , & Seisdedos, N. (2015). Cuestionario de Ansiedad Estado‐Rasgo (9th edn.). TEA Ediciones.

[brb32624-bib-0014] Burokas, A. , Moloney, R. D. , Dinan, T. G. , & Cryan, J. F. (2015). Microbiota regulation of the mammalian gut‐brain axis. Advances in Applied Microbiology, 91, 1–62. 10.1016/bs.aambs.2015.02.001 25911232

[brb32624-bib-0015] Butler, M. I. , Cryan, J. F. , & Dinan, T. G. (2019). Man and the microbiome: A new theory of everything? Annual Review of Clinical Psychology, 15(1), 371–398. 10.1146/annurev-clinpsy-050718-095432 30786244

[brb32624-bib-0016] Butnoriene, J. , Bunevicius, A. , Saudargiene, A. , Nemeroff, C. B. , Norkus, A. , Ciceniene, V. , & Bunevicius, R. (2015). Metabolic syndrome, major depression, generalized anxiety disorder, and ten‐year all‐cause and cardiovascular mortality in middle aged and elderly patients. International Journal of Cardiology, 190, 360–366. 10.1016/j.ijcard.2015.04.122 25939128

[brb32624-bib-0017] Cohen, A. , Colodner, R. , Masalha, R. , & Haimov, I. (2019). The relationship between tobacco smoking, cortisol secretion, and sleep continuity. Substance Use & Misuse, 54(10), 1705–1714. 10.1080/10826084.2019.1608250 31081433

[brb32624-bib-0018] Coit, P. , & Sawalha, A. H. (2016). The human microbiome in rheumatic autoimmune diseases: A comprehensive review. Clinical Immunology, 170, 70–79. 10.1016/j.clim.2016.07.026 27493014

[brb32624-bib-0019] Craske, M. G. , & Lewin, M. R. (2007). Trastorno por pánico. En Manual para el tratamiento cognitivo‐conductual de los trastornos psicológicos. Trastornos por ansiedad, sexuales, afectivos y psicóticos (Vol. 1). Siglo XXI.

[brb32624-bib-0020] Cryan, J. F. , O'Riordan, K. J. , Cowan, C. S. M. , Sandhu, K. V. , Bastiaanssen, T. F. S. , Boehme, M. , Codagnone, M. G. , Cussotto, S. , Fulling, C. , Golubeva, A. V. , Guzzetta, K. E. , Jaggar, M. , Long‐Smith, C. M. , Lyte, J. M. , Martin, J. A. , Molinero‐Perez, A. , Moloney, G. , Morelli, E. , Morillas, E. , … Dinan, T. G. (2019). The microbiota‐gut‐brain axis. Physiological Reviews, 99(4), 1877–2013. 10.1152/physrev.00018.2018 31460832

[brb32624-bib-0021] De Gregori, M. , Belfer, I. , De Giorgio, R. , Marchesini, M. , Muscoli, C. , Rondanelli, M. , Martini, D. , Mena, P. , Arranz, L. I. , Lorente‐Cebrián, S. , Perna, S. , Villarini, A. , Salamone, M. , Allegri, M. , & Schatman, M. E. (2018). Second edition of SIMPAR's “Feed Your Destiny” workshop: The role of lifestyle in improving pain management. Journal of Pain Research, 11, 1627–1636. 10.2147/JPR.S160660 30214272 PMC6118253

[brb32624-bib-0022] Dimenäs, E. , Glise, H. , Hallerbäck, B. , Hernqvist, H. , Svedlund, J. , & Wiklund, I. (1995). Well‐being and gastrointestinal symptoms among patients referred to endoscopy owing to suspected duodenal ulcer. Scandinavian Journal of Gastroenterology, 30(11), 1046–1052. 8578162 10.3109/00365529509101605

[brb32624-bib-0023] Dinan, T. G. , & Cryan, J. F. (2013). Melancholic microbes: A link between gut microbiota and depression? Neurogastroenterology & Motility, 25(9), 713–719. 10.1111/nmo.12198 23910373

[brb32624-bib-0024] Fekedulegn, D. B. , Andrew, M. E. , Burchfiel, C. M. , Violanti, J. M. , Hartley, T. A. , Charles, L. E. , & Miller, D. B. (2007). Area under the curve and other summary indicators of repeated waking cortisol measurements. Psychosomatic Medicine, 69(7), 651–659. 10.1097/PSY.0b013e31814c405c 17766693

[brb32624-bib-0025] Fiksdal, A. , Hanlin, L. , Kuras, Y. , Gianferante, D. , Chen, X. , Thoma, M. V. , & Rohleder, N. (2019). Associations between symptoms of depression and anxiety and cortisol responses to and recovery from acute stress. Psychoneuroendocrinology, 102, 44–52. 10.1016/j.psyneuen.2018.11.035 30513499 PMC6420396

[brb32624-bib-0026] Forsythe, P. , Sudo, N. , Dinan, T. , Taylor, V. H. , & Bienenstock, J. (2010). Mood and gut feelings. Brain, Behavior, and Immunity, 24(1), 9–16. 10.1016/j.bbi.2009.05.058 19481599

[brb32624-bib-0027] Foster, J. A. , & McVey Neufeld, K. ‐A. (2013). Gut‐brain axis: How the microbiome influences anxiety and depression. Trends in Neurosciences, 36(5), 305–312. 10.1016/j.tins.2013.01.005 23384445

[brb32624-bib-0028] Foster, J. A. , Rinaman, L. , & Cryan, J. F. (2017). Stress & the gut‐brain axis: Regulation by the microbiome. Neurobiology of Stress, 7, 124–136. 10.1016/j.ynstr.2017.03.001 29276734 PMC5736941

[brb32624-bib-0029] Ganci, M. , Suleyman, E. , Butt, H. , & Ball, M. (2019). The role of the brain–gut–microbiota axis in psychology: The importance of considering gut microbiota in the development, perpetuation, and treatment of psychological disorders. Brain and Behavior, 9(11), e01408. 10.1002/brb3.1408 31568686 PMC6851798

[brb32624-bib-0030] Giollabhui, N. M. , Swistun, D. , Murray, S. , Moriarity, D. P. , Kautz, M. M. , Ellman, L. M. , Olino, T. M. , Coe, C. L. , Abramson, L. Y. , & Alloy, L. B. (2020). Executive dysfunction in depression in adolescence: The role of inflammation and higher body mass. Psychological Medicine, 50(4), 683–691. 10.1017/S0033291719000564 30919789 PMC6765453

[brb32624-bib-0031] Haro, J. M. , Palacín, C. , Vilagut, G. , Martínez, M. , Bernal, M. , Luque, I. , Codony, M. , Dolz, M. , & Alonso, J. (2006). Prevalencia de los trastornos mentales y factores asociados: Resultados del estudio ESEMeD‐España. Medicina clínica, 126(12), 445–451. 16620730 10.1157/13086324

[brb32624-bib-0032] Jeon, S. W. , Yoon, H. ‐K. , & Kim, Y. ‐K. (2019). Role of inflammation in psychiatric disorders. In Y.‐K. Kim (Ed.), Frontiers in psychiatry. Artificial Intelligence, Precision Medicine, and Other Paradigm Shifts Advances in Experimental Medicine and Biology (Vol. 1192, pp. 491–501). Springer. 10.1007/978-981-32-9721-0_24 31705510

[brb32624-bib-0033] Johnson, B. M. , Gaudreau, M. ‐C. , Gudi, R. , Brown, R. , Gilkeson, G. , & Vasu, C. (2020). Gut microbiota differently contributes to intestinal immune phenotype and systemic autoimmune progression in female and male lupus‐prone mice. Journal of Autoimmunity, 108, 102420. 10.1016/j.jaut.2020.102420 32019684 PMC7204266

[brb32624-bib-0034] Khlevner, J. , Park, Y. , & Margolis, K. G. (2018). Brain–gut axis. Gastroenterology Clinics of North America, 47(4), 727–739. 10.1016/j.gtc.2018.07.002 30337029 PMC6829582

[brb32624-bib-0035] Komine, M. (2020). Recent advances in psoriasis research; the clue to mysterious relation to gut microbiome. International Journal of Molecular Sciences, 21(7), 2582. 10.3390/ijms21072582 PMC7177330 32276410

[brb32624-bib-0036] Kulich, K. , Piqué, J. , Vegazo, O. , Jiménez, J. , Zapardiel, J. , Carlsson, J. , & Wiklund, I. (2005). Validación psicométrica de la traducción al español de la escala de evaluación de síntomas gastrointestinales (GSRS) y del cuestionario de calidad de vida de reflujo y dispepsia (QOLRAD) en los pacientes con enfermedad por reflujo gastroesofágico. Revista Clinica Espanola, 205(12), 588–594. 16527180 10.1016/s0014-2565(05)72651-5

[brb32624-bib-0037] Lange, K. W. , Lange, K. M. , Nakamura, Y. , & Kanaya, S. (2020). Is there a role of gut microbiota in mental health? Journal of Food Bioactives, 9, 4–9. 10.31665/JFB.2020.9213

[brb32624-bib-0038] Li, N. , Wang, Q. , Wang, Y. , Sun, A. , Lin, Y. , Jin, Y. , & Li, X. (2019). Fecal microbiota transplantation from chronic unpredictable mild stress mice donors affects anxiety‐like and depression‐like behavior in recipient mice via the gut microbiota‐inflammation‐brain axis. Stress (Amsterdam, the Netherlands), 22(5), 592–602. 10.1080/10253890.2019.1617267 31124390

[brb32624-bib-0039] Lopresti, A. L. (2017). Cognitive behaviour therapy and inflammation: A systematic review of its relationship and the potential implications for the treatment of depression. Australian & New Zealand Journal of Psychiatry, 51(6), 565–582. 10.1177/0004867417701996 28382827

[brb32624-bib-0040] Luetters, C. , Huang, M. ‐H. , Seeman, T. , Buckwalter, G. , Meyer, P. M. , Avis, N. E. , Sternfeld, B. , Johnston, J. M. , & Greendale, G. A. (2007). Menopause transition stage and endogenous estradiol and follicle‐stimulating hormone levels are not related to cognitive performance: Cross‐sectional results from the Study of Women's Health across the Nation (SWAN). Journal of Women's Health, 16(3), 331–344. 10.1089/jwh.2006.0057 17439378

[brb32624-bib-0041] Majd, M. , Saunders, E. F. H. , & Engeland, C. G. (2020). Inflammation and the dimensions of depression: A review Frontiers in Neuroendocrinology, 56, 100800. 10.1016/j.yfrne.2019.100800 31654681 PMC13105217

[brb32624-bib-0042] Manigault, A. W. , Shorey, R. C. , Hamilton, K. , Scanlin, M. C. , Woody, A. , Figueroa, W. S. , France, C. R. , & Zoccola, P. M. (2019). Cognitive behavioral therapy, mindfulness, and cortisol habituation: A randomized controlled trial. Psychoneuroendocrinology, 104, 276–285. 10.1016/j.psyneuen.2019.03.009 30917336

[brb32624-bib-0043] Mayer, E. A. (2011). Gut feelings: The emerging biology of gut–brain communication. Nature Reviews Neuroscience, 12(8), 453. 10.1038/nrn3071 21750565 PMC3845678

[brb32624-bib-0044] Meuret, A. E. , Tunnell, N. , & Roque, A. (2020). Anxiety disorders and medical comorbidity: Treatment implications. In Y. ‐K. Kim (Ed.), Anxiety disorders: Rethinking and understanding recent discoveries (pp. 237–261). Springer. 10.1007/978-981-32-9705-0_15 32002933

[brb32624-bib-0045] Michopoulos, V. , Powers, A. , Gillespie, C. F. , Ressler, K. J. , & Jovanovic, T. (2017). Inflammation in fear‐and anxiety‐based disorders: PTSD, GAD, and beyond. Neuropsychopharmacology, 42(1), 254. 27510423 10.1038/npp.2016.146 PMC5143487

[brb32624-bib-0046] Milrad, S. F. , Hall, D. L. , Jutagir, D. R. , Lattie, E. G. , Czaja, S. J. , Perdomo, D. M. , Fletcher, M. A. , Klimas, N. , & Antoni, M. H. (2018). Depression, evening salivary cortisol and inflammation in chronic fatigue syndrome: A psychoneuroendocrinological structural regression model. International Journal of Psychophysiology, 131, 124–130. 10.1016/j.ijpsycho.2017.09.009 28918107 PMC5851813

[brb32624-bib-0047] Moreno, P. , & Martín, J. C. (2011). *Tratamiento psicológico del trastorno de pánico y la agorafobia: Manual para terapeutas*. Desclée de Brouwer.

[brb32624-bib-0048] Niles, A. N. , Smirnova, M. , Lin, J. , & O'Donovan, A. (2018). Gender differences in longitudinal relationships between depression and anxiety symptoms and inflammation in the health and retirement study. Psychoneuroendocrinology, 95, 149–157. 10.1016/j.psyneuen.2018.05.035 29864671 PMC6354934

[brb32624-bib-0049] Ouabbou, S. , He, Y. , Butler, K. , & Tsuang, M. (2020). Inflammation in mental disorders: Is the microbiota the missing link? Neuroscience Bulletin, 36(9), 1071–1084. 10.1007/s12264-020-00535-1 32592144 PMC7475155

[brb32624-bib-0050] Paiva, I. H. R. , Duarte‐Silva, E. , & Peixoto, C. A. (2020). The role of prebiotics in cognition, anxiety, and depression. European Neuropsychopharmacology, 34, 1–18. 10.1016/j.euroneuro.2020.03.006 32241688

[brb32624-bib-0051] Peirce, J. M. , & Alviña, K. (2019). The role of inflammation and the gut microbiome in depression and anxiety. Journal of Neuroscience Research, 97(10), 1223–1241. 10.1002/jnr.24476 31144383

[brb32624-bib-0052] Petrovsky, N. , McNair, P. , & Harrison, L. C. (1998). Diurnal rhythms of pro‐inflammatory cytokines: Regulation by plasma cortisol and therapeutic implications Cytokine, 10(4), 307–312. 10.1006/cyto.1997.0289 9617577

[brb32624-bib-0053] Petrowski, K. , Wichmann, S. , & Kirschbaum, C. (2018). Stress‐induced pro‐ and anti‐inflammatory cytokine concentrations in panic disorder patients. Psychoneuroendocrinology, 94, 31–37. 10.1016/j.psyneuen.2018.05.005 29754003

[brb32624-bib-0054] Powell, D. J. , & Schlotz, W. (2012). Daily life stress and the cortisol awakening response: Testing the anticipation hypothesis. Plos One, 7(12), e52067. 10.1371/journal.pone.0052067 23284871 PMC3527370

[brb32624-bib-0055] Pruessner, J. C. , Kirschbaum, C. , Meinlschmid, G. , & Hellhammer, D. H. (2003). Two formulas for computation of the area under the curve represent measures of total hormone concentration versus time‐dependent change. Psychoneuroendocrinology, 28(7), 916–931. 10.1016/S0306-4530(02)00108-7 12892658

[brb32624-bib-0056] Qing, H. , Desrouleaux, R. , Israni‐Winger, K. , Mineur, Y. S. , Fogelman, N. , Zhang, C. , Rashed, S. , Palm, N. W. , Sinha, R. , Picciotto, M. R. , Perry, R. J. , & Wang, A. (2020). Origin and function of stress‐induced IL‐6 in murine models. Cell, 182(2), 372–387. 10.1016/j.cell.2020.05.054 32610084 PMC7384974

[brb32624-bib-0057] Quagliato, L. A. , & Nardi, A. E. (2018). Cytokine alterations in panic disorder: A systematic review. Journal of Affective Disorders, 228, 91–96. 10.1016/j.jad.2017.11.094 29241050

[brb32624-bib-0058] Raff, H. , & Levitzky, M. (2013). Fisiología médica. Un enfoque por aparatos y sistemas (1st edn.). McGraw‐Hill Interamericana.

[brb32624-bib-0059] Rea, K. , Dinan, T. G. , & Cryan, J. F. (2019). Gut microbiota: A perspective for psychiatrists. Neuropsychobiology, 79(1), 50–62. 10.1159/000504495 31726457

[brb32624-bib-0060] Remor, E. , & Carrobles, J. A. (2001). Versión Española de la Escala de Estrés Percibido (PSS‐14): Estudio psicométrico en una muestra VIH+. [Spanish version of the Perceived Stress Scale (PSS‐14): Psychometric study in a HIV+ sample.]. Ansiedad y Estrés, 7(2‐3), 195–201.

[brb32624-bib-0061] Richards, C. (2018). *Impacts of early life adversity on microbiota and immune functioning in individuals with major depressive disorder* [Master's thesis, Carleton University]. 10.22215/etd/2018-13304

[brb32624-bib-0062] Rook, G. A. W. , Raison, C. L. , & Lowry, C. A. (2014). Microbiota, immunoregulatory old friends and psychiatric disorders. In M. Lyte, & J. F. Cryan (Eds.), Microbial endocrinology: The microbiota‐gut‐brain axis in health and disease (Vol. 817, pp. 319–356). Springer. 10.1007/978-1-4939-0897-4_15 24997041

[brb32624-bib-0063] Salim, S. , Chugh, G. , & Asghar, M. (2012). Inflammation in anxiety. Advances in Protein Chemistry and Structural Biology, 88, 1–25. 10.1016/B978-0-12-398314-5.00001-5 22814704

[brb32624-bib-0064] Sanz, J. , García‐Vera, M. P. , Espinosa, R. , Fortún, M. , & Vázquez, C. (2005). Adaptación española del Inventario para la Depresión de Beck‐II (BDI‐II): 3. Propiedades psicométricas en pacientes con trastornos psicológicos. Clínica y Salud, 16, 23.

[brb32624-bib-0065] Sarkar, A. , Harty, S. , Lehto, S. M. , Moeller, A. H. , Dinan, T. G. , Dunbar, R. I. M. , Cryan, J. F. , & Burnet, P. W. J. (2018). The Microbiome in psychology and cognitive neuroscience. Trends in Cognitive Sciences, 22(7), 611–636. 10.1016/j.tics.2018.04.006 29907531

[brb32624-bib-0066] Schmidt, C. (2015). Mental health: Thinking from the gut. Nature, 518(s7540), S12–S15. 10.1038/518S13a 25715275

[brb32624-bib-0067] Schnorr, S. L. , & Bachner, H. A. (2016). Integrative therapies in anxiety treatment with special emphasis on the gut microbiome. The Yale Journal of Biology and Medicine, 89(3), 397–422. 27698624 PMC5045149

[brb32624-bib-0068] Stalder, T. , Kirschbaum, C. , Kudielka, B. M. , Adam, E. K. , Pruessner, J. C. , Wüst, S. , Dockray, S. , Smyth, N. , Evans, P. , Hellhammer, D. H. , Miller, R. , Wetherell, M. A. , Lupien, S. J. , & Clow, A. (2016). Assessment of the cortisol awakening response: Expert consensus guidelines. Psychoneuroendocrinology, 63, 414–432. 10.1016/j.psyneuen.2015.10.010 26563991

[brb32624-bib-0069] Steptoe, A. , & Serwinski, B. (2016). Cortisol awakening response. In G. Fink (Ed.), Stress: Concepts, cognition, emotion, and behavior (pp. 277–283). Elsevier. 10.1016/B978-0-12-800951-2.00034-0

[brb32624-bib-0070] Takada, M. , Nishida, K. , Kataoka‐Kato, A. , Gondo, Y. , Ishikawa, H. , Suda, K. , Kawai, M. , Hoshi, R. , Watanabe, O. , Igarashi, T. , Kuwano, Y. , Miyazaki, K. , & Rokutan, K. (2016). Probiotic *Lactobacillus casei* strain Shirota relieves stress‐associated symptoms by modulating the gut‐brain interaction in human and animal models. Neurogastroenterology and Motility, 28(7), 1027–1036. 10.1111/nmo.12804 26896291

[brb32624-bib-0071] Tao, H. , Wang, C. ‐R. , Guo, J. ‐C. , & Guo, M. (2020). Research progress on the relationship between intestinal flora and mental and psychological diseases. Advances in Microbiology, 10(06), 295–305. 10.4236/aim.2020.106021

[brb32624-bib-0072] Tükel, R. , Arslan, B. A. , Ertekin, B. A. , Ertekin, E. , Oflaz, S. , Ergen, A. , Kuruca, S. E. , & İsbir, T. (2012). Decreased IFN‐γ and IL‐12 levels in panic disorder. Journal of Psychosomatic Research, 73(1), 63–67. 10.1016/j.jpsychores.2012.04.012 22691562

[brb32624-bib-0073] Virili, C. , Fallahi, P. , Antonelli, A. , Benvenga, S. , & Centanni, M. (2018). Gut microbiota and Hashimoto's thyroiditis. Reviews in Endocrine and Metabolic Disorders, 19(4), 293–300. 10.1007/s11154-018-9467-y 30294759

[brb32624-bib-0074] Vreeburg, S. A. , Zitman, F. G. , van Pelt, J. , DeRijk, R. H. , Verhagen, J. C. M. , van Dyck, R. , Hoogendijk, W. J. G. , Smit, J. H. , & Penninx, B. W. J. H. (2010). Salivary cortisol levels in persons with and without different anxiety disorders. Psychosomatic Medicine, 72(4), 340–347. 10.1097/PSY.0b013e3181d2f0c8 20190128

[brb32624-bib-0075] Wang, S. , Harvey, L. , Martin, R. , van der Beek, E. M. , Knol, J. , Cryan, J. F. , & Renes, I. B. (2018). Targeting the gut microbiota to influence brain development and function in early life. Neuroscience & Biobehavioral Reviews, 95, 191–201. 10.1016/j.neubiorev.2018.09.002 30195933

[brb32624-bib-0076] World Health Organization (WHO) . (2000). *Guia de bolsillo de la clasificación CIE‐10: Clasificación de los trastornos mentales y del comportamiento*. Editorial Médica Panamericana. https://apps.who.int/iris/handle/10665/42326

[brb32624-bib-0077] Yang, C. ‐J. , Liu, D. , Xu, Z. ‐S. , Shi, S. ‐X. , & Du, Y. ‐J. (2017). The pro‐inflammatory cytokines salivary cortisol and alpha‐amylase are associated with generalized anxiety disorder (GAD) in patients with asthma. Neuroscience Letters, 656, 15–21. 10.1016/j.neulet.2017.07.021 28716529

[brb32624-bib-0078] Zainal, N. H. , & Newman, M. G. (2022). Inflammation mediates depression and generalized anxiety symptoms predicting executive function impairment after 18 years. Journal of Affective Disorders, 296, 465–475. 10.1016/j.jad.2021.08.077 34649180 PMC8603378

